# Dissecting the Root Nodule Transcriptome of Chickpea (*Cicer arietinum* L.)

**DOI:** 10.1371/journal.pone.0157908

**Published:** 2016-06-27

**Authors:** Chandra Kant, Seema Pradhan, Sabhyata Bhatia

**Affiliations:** National Institute of Plant Genome Research, Aruna Asaf Ali Marg, Post Box No. 10531, New Delhi 110067, India; National Institute of Plant Genome Research (NIPGR), INDIA

## Abstract

A hallmark trait of chickpea (*Cicer arietinum* L.), like other legumes, is the capability to convert atmospheric nitrogen (N_2_) into ammonia (NH_3_) in symbiotic association with *Mesorhizobium ciceri*. However, the complexity of molecular networks associated with the dynamics of nodule development in chickpea need to be analyzed in depth. Hence, in order to gain insights into the chickpea nodule development, the transcriptomes of nodules at early, middle and late stages of development were sequenced using the Roche 454 platform. This generated 490.84 Mb sequence data comprising 1,360,251 reads which were assembled into 83,405 unigenes. Transcripts were annotated using Gene Ontology (GO), Cluster of Orthologous Groups (COG) and Kyoto Encyclopedia of Genes and Genomes (KEGG) metabolic pathways analysis. Differential expression analysis revealed that a total of 3760 transcripts were differentially expressed in at least one of three stages, whereas 935, 117 and 2707 transcripts were found to be differentially expressed in the early, middle and late stages of nodule development respectively. MapMan analysis revealed enrichment of metabolic pathways such as transport, protein synthesis, signaling and carbohydrate metabolism during root nodulation. Transcription factors were predicted and analyzed for their differential expression during nodule development. Putative nodule specific transcripts were identified and enriched for GO categories using BiNGO which revealed many categories to be enriched during nodule development, including transcription regulators and transporters. Further, the assembled transcriptome was also used to mine for genic SSR markers. In conclusion, this study will help in enriching the transcriptomic resources implicated in understanding of root nodulation events in chickpea.

## Introduction

Symbiotic nitrogen fixation (SNF) is an important biological event that allows legumes to grow efficiently under nitrogen limiting conditions and also has important agronomical and environmental benefits. Due to their unique ability to form symbiotic relationship with a group of nitrogen fixing bacteria called ‘Rhizobia’, legumes represent an important and diverse group of plants since 50–70% of biological nitrogen fixation, leading to a terrestrial input of 40–50 million tons of nitrogen per year [1], is carried out by symbiotic nitrogen fixation. Chickpea (*C*. *arietinum* L.) 2n = 16, is one of the most important staple legume crops widely grown across many semi-arid regions of the world including the Indian subcontinent and has the capacity to fix large quantities of atmospheric nitrogen by forming a symbiotic interaction with *Mesorhizobium ciceri*. This process of symbiosis and nodulation leading to N2 fixation is quite complex and tightly regulated, but very scantily understood at the molecular level.

Initiation of root nodulation occurs when (Nodulation) Nod factor (NF) signals secreted by the rhizobia are perceived by root hairs, which initiate curling of root hair followed by initiation of cell division and nodule primordium formation which finally develops into the new organ called ‘nodule’. Here the bacteria differentiate to form bacteroids and fix atmospheric N_2_ to ammonia for direct use of the plant. In return the plant serves as a carbon source for the bacteroids. At a cellular level, the NF perception is characterized by calcium spiking and induction of NF induced genes.

Some of the genes involved at different steps in root nodule formation have been characterized in *L*. *japonicus*, *Medicago truncatula* and *G*. *max*. LjNFR1, LjNFR5 and MtDMI2/NORK are known to be involved in perception of NOD factor signals by the root hair [2–4]. A number of genes such as LjCASTOR, LjPOLLUX [5], MtDMI1[6], nucleoporins LjNUP85 and LjNUP133 [7, 8], CCaMK encoded by MtDMI3 and LjCYCLOPS [9] are known to be involved in calcium spiking and related signaling. Further, a number of genes downstream of the NOD factor signaling pathway, including several transcription factors such as MtNSP1[10], MtNSP2[11], LjNSP1, LjNSP2 [12], MtHAP2 [13] and LjNIN [14] have been characterized and shown to be involved in regulation of genes expressed during various stages of nodule development.

Considerable progress has been made in the last few years in legume genomics, notably by sequencing the genomes of legumes such as *L*. *japonicus* [15], *G*. *max* [16], *M*. *truncatula*[17], *Cajanus cajan* [18] and *Cicer arietinum* [19, 20]. A number of transcriptome analyses have also been carried out using microarray and next generation sequencing (NGS) technologies such as Roche/454, Illumina/Solexa and ABI/SOLiD and have provided deep insights into the transcriptional complexity of the organism [21, 22]. The Roche/454 Genome Sequencer FLX platform has been shown to provide an improved coverage of transcriptome as compared to conventional methods of EST sequencing [23] and has been used in studies involving differential expression analysis [24, 25].

In the recent decade, many studies reporting the high throughput transcriptome analysis of root nodules in different legumes, especially the model legumes have become available. For example, analysis in *M*. *truncatula* showed differential expression of genes in early stage of root nodulation [26]. Expression profiling in *M*. *truncatula* showed that more than 750 genes are differentially expressed during nodulation events [27]. Another analysis in *M*. *truncatula* revealed the expression profiles of putative transcriptional regulators that orchestrate developmental programs during nodulation [28]. In *L*. *japonicus*, transcriptome analysis using SAGE showed 407 tags expressed in significantly higher amount in nodule when compared to root [29]. Global analysis of the transcriptome in wild type and mutant *L*. *japonicus* at different stages of symbiotic interactions revealed a large number of transcripts predicted to encode transcriptional regulators, receptors and proteins involved in signal transduction. Moreover many genes of unknown function were found to be regulated during nodule organogenesis [30]. In crop legumes such as soybean, 1,973 genes were found differentially expressed during nodulation using 3 different technologies i.e. microarray hybridization, Illumina sequencing and quantitative real time RT PCR [31]. A genome based deep SuperSAGE study of mature root nodules of chickpea has been performed recently [32] that showed only 71 genes to be differentially expressed in root nodules. Hence, a high throughput, in depth analysis of the chickpea root nodule was desirable for gaining deeper insights into the overall transcriptional complexity of nodulation events. In this study, deep sequencing of the transcriptomes of nodules at various stages of development was undertaken, the transcriptomes were assembled and annotated and further analyzed to identify genes which were differentially expressed at various stages of root nodule development. Further nodule-specific transcripts were also identified. Moreover, significantly enriched transcriptional regulators and metabolic pathways were identified to provide insights into the various processes regulating chickpea root nodule development.

## Materials and Methods

### Generation of nodule tissue

Chickpea seeds (*Cicer arietinum* cv. BG256) were surface sterilized using 70% ethanol (30 seconds), followed by 0.1% HgCl_2_ (2 mins) and then rinsed in milli-Q water 4–5 times. Seeds were soaked overnight in water and then allowed to germinate on an agar plate for 2–3 days. *M*. *ciceri* was grown in Yeast Mannitol Broth (YMB) medium. Seeds were given infection by dipping them in *M*. *ciceri* culture. Seeds were grown on sterilized sand and provided with nitrogen free McKnight’s solution (McKnight, 1949), twice a week. Infected roots were harvested at 3 HPI (Hours post infection), 6 HPI, 12 HPI, 24 HPI, 36 HPI, 2 DPI (Days post infection) and daily thereafter upto 28days. Three biological replicates of each tissue sample were pooled into 3 groups i.e. 3HPI-36HPI, 2DPI-10DPI and 11DPI-28DPI, and used as experimental tissue. Correspondingly, uninfected roots (without nodules) at the same stages of development as nodules, was also collected and used as control tissue.

### RNA isolation and sequencing of cDNA libraries

Total RNA was isolated from the nodulating and non-nodulating root tissue by using LiCl precipitation method and a quality check was carried out on the bioanalyser as described by Pradhan et al. [25]. mRNA was purified from total RNA samples using PolyATtract mRNA Isolation System according to the manufacturer’s instructions (Promega Corporation, Madison, WI, USA). Four cDNA libraries (3 libraries of nodulating tissue at various stages and one control) were generated using the Universal RiboClone cDNA Synthesis System (Promega Corporation, Madison, WI, USA). Double-stranded cDNA was synthesized using the Universal RiboClone cDNA Synthesis System (Promega Corporation, Madison, WI, USA) using random hexameric primers and following the manufacturer’s protocol. All cDNA libraries were sequenced using the Roche GS FLX Titanium series sequencing reagents and sequencer. A stringent quality filtering using the NGS ToolKit [33] was done with average quality score of all the reads above 30, with cut off phred quality score of 20 for over 70% of individual read length. Reads were aligned to genome of *M*. *ciceri* [34] (ASM18590v1) using BLASTN with e-value 1E-05, and reads mapped to *M*. *ciceri* genome were discarded.

### Assembly and annotation

The high quality reads were mapped onto the reference genome using gsMapper and assembled using Newbler 2.5.3 with default parameters. Assembled sequences were further clustered using CAP3 [35]. To assign putative function to chickpea transcripts, they were subjected to BLASTX search against annotated protein sequences present in UniProtKB/Swiss-Prot database (http://www.uniprot.org/downloads). E-value ≤1E-05 was used to select the best BLASTX hit. The GO slim terms for molecular function, biological process and cellular component categories associated with BLASTX hit of Uniprot protein sequences were assigned using perl script, to corresponding chickpea transcript. Chickpea transcripts were aligned with COG database using BLASTX at E-value ≤1E-05 and were annotated using perl script. KEGG pathways annotation was done online by KAAS (http://www.genome.jp/tools/kaas/) using single directional best hit (SBH) method. To identify transcription factors, plant transcription factor database (planttfdb.cbi.pku.edu.cn) was downloaded. Hidden Markov Model (HMM) profiles were built for each TF family and used in HMMsearch at E-value ≤1E-05. A microsatellite search program MISA (http://pgrc.ipkgatersleben.de/misa/) was used to identify microsatellite motifs. All types of simple sequence repeats (SSRs) were searched ranging from dinucleotide to hexanucleotides using the following parameters: perfect repeats of dinucleotides ≥ 6 repeats, trinucleotides ≥ 4 repeats, tetranucleotides ≥ 3 repeats, pentanucleotides and hexanucleotides ≥ 3 repeats. Primers were designed from the flanking sequences of SSRs, using BatchPrimer3 v1.0 (http://probes.pw.usda.gov/batchprimer3/).

### Identification of nodule specific transcripts

In order to identify putative nodule specific transcripts, the assembled root nodule transcripts were compared with the publicaly available chickpea transcripts from all other tissues of chickpea such as which includes shoot, root, mature leaf, flower bud and young pod [36] and seed [25]. A TBLASTX analysis was done at E-value ≤1E-05 to align transcripts. Nodule transcripts which did not find matches with any of the other tissue transcripts were considered as putative nodule specific transcripts. These putative nodule specific transcripts were annotated by using Non Redundant (NR) database of NCBI in Blast2GO. Further, they were subjected to GO enrichment by using BiNGO 2.44 [37], in Cytoscape 2.8.3.

### Differential expression analysis

Filtered reads of each stage were mapped onto the combined assembly using gsMapper 2.5.3 with default parameters. Reads Per Kilobase of transcript per Million mapped reads (RPKM) [38] values were calculated using formula RPKM = [(Number of mapping reads)*1000*10^9^/(length of transcript)(number of total reads)]. The heat map was generated based on the log_2_-transformed RPKM values using MeV v. 4.9 (http://www.tm4.org/mev.html), representing the expression profile of transcripts. Differential expression of genes was calculated using DEGseq package [39] in R, at p-value cut-off of 0.005 after Benjamini Hochberg adjustment [40]. To investigate the role of differentially expressed transcripts in metabolic pathways, a BLASTX search was done with *M*. *truncatula* peptides at E-value ≤1E-05, downloaded from Phytozome 9.1 (ftp://ftp.jgi-psf.org/pub/compgen/phytozome/v9.0/Mtruncatula/annotation/) and metabolic pathways were searched using MapMan [41].

### Quantitative Real-Time PCR (qRT-PCR) analysis

Quantitative real time PCR analysis was performed to generate the expression profile of some genes in a number of tissues. Total RNA was isolated from 3HPI, 6HPI, 12HPI, 24HPI, 3DPI, 7DPI, 14DPI, mature nodule (21 DPI), uninfected root and shoot form 7 day old seedling, using LiCl extraction method. Integrity of RNA was checked on 1.2% denaturing agarose gel. First strand cDNA was made using iScript^tm^kit (BIO-RAD) according to manufacturer’s protocol. Primers were designed using Primer Express® Software v3.0.1 (Life technologies). Quantitative real time PCR was performed using Fast SYBR Green (Applied Biosystems) master mix on a ViiA7 (Applied Biosystems) machine. EF-1**α** was used as internal control [42]. Fold changes were calculated using (RQ = 2^-ΔΔCT^) formula. Melting curves were analysed to see the homogeneity of the product formed in PCR reaction.

## Results

### Sequencing and assembly of chickpea root nodule transcriptome

Four cDNA libraries were constructed from three stages of developing chickpea root nodule (3HPI-36HPI (Hours post infection), 2DPI-10DPI (Days post infection) and 11DPI-28DPI) as well as from non-infected chickpea root (Control) and sequenced on the Roche 454 GS FLX Titanium platform. The sequence data obtained was deposited in the SRA database under the accession numbers SRX330836, SRX330835, SRX330827 and SRX330813. A total of 1,360,251 reads (330,667 in uninfected root and 1,029,584 in root nodule) were generated amounting to 490.84Mb of sequence data (119.62 Mb in uninfected root and 371.22 Mb in root nodule). The average read length was 360.84 bases. After stringent quality filtering using the NGS ToolKit [33], 1,235,093 high quality reads (465.85 Mb) were obtained with average read length of 377 bases, accounting for 90.79% of the total raw reads (Table 1). A read distribution statistics showed that 81% of the filtered reads were larger than 300 bases. Alignment of these raw reads to *M*. *ciceri* genome [34] (ASM18590v1) resulted in further filtering of 11,680 reads.

**Table 1:** Summary of sequenced reads generated from control root and developing stages of root nodules.

Tissue	Number of reads	High quality reads (%)	Longest read length	Average read length
Control root	330,667	90.48	611	377
3HPI-36HPI	180,272	88.80	600	363
2DPI-10DPI	324,289	91.02	650	388
11DPI-28DPI	525,023	89.33	699	373
Total	1,360,251	89.94	699	377

Using Newbler v. 2.5.3, a total of 1,144,349 (93.53%) high quality reads representing 415.41 Mb were assembled into 87,342 contigs. These contigs were further assembled using the cap3 program which resulted in 83,405 unigenes with an average size of 388 bases with N50 value of 496 bases and N50 Index value of 18,391(Table 2; S1 Data; S1 Fig). This set of 83,405 unigenes were used to perform a BLASTN search against the available chickpea gene models from the whole genome sequence of chickpea [19] which resulted in alignment of 60,360 unigenes onto 20,406 gene models whereas 23,045 unigenes could not be aligned. These 23,045 unaligned unigenes were mapped onto the chickpea genome sequence, which revealed alignment of 22,693 unigenes with the chickpea genome. Some of these unigenes were validated by PCR amplification to ensure their presence in the transcriptome (S2 Fig). Comparison of unigenes with the Non-redundant (Nr) protein database of NCBI (ftp://ftp.ncbi.nlm.nih.gov) showed that 75.16% found a match while 20,711 did not find a match. Further, the assembly was validated by aligning the assembled transcripts with some of the known proteomes of legumes (*G*.*max*, *L*. *japonicus* and *M*. *truncatula*) and model plants available at phytozome (http://www.phytozome.net/) such as *Arabidopsis thaliana* and *Populus trichocarpa*. BLASTX search against proteome databases showed alignment of maximum number of chickpea nodule transcripts to the proteome of *G*. *max* (69.84%) followed by *P*. *trichocarpa* (62.98%), *M*. *truncatula* (62.31%) *and L*. *japonicus* (61.27%). As expected, least number of chickpea transcripts could be aligned to the proteome of *A*. *thaliana* (59.69%) (S1 Table).

**Table 2:** Assembly statistics.

Total number of contigs	83,405
Total number of bases	32,393,843
N50 value	496
N50 Index value	18,391
Average length of contigs	388
Largest length of contigs	8,483

### Functional Annotation

**Fig 1:**
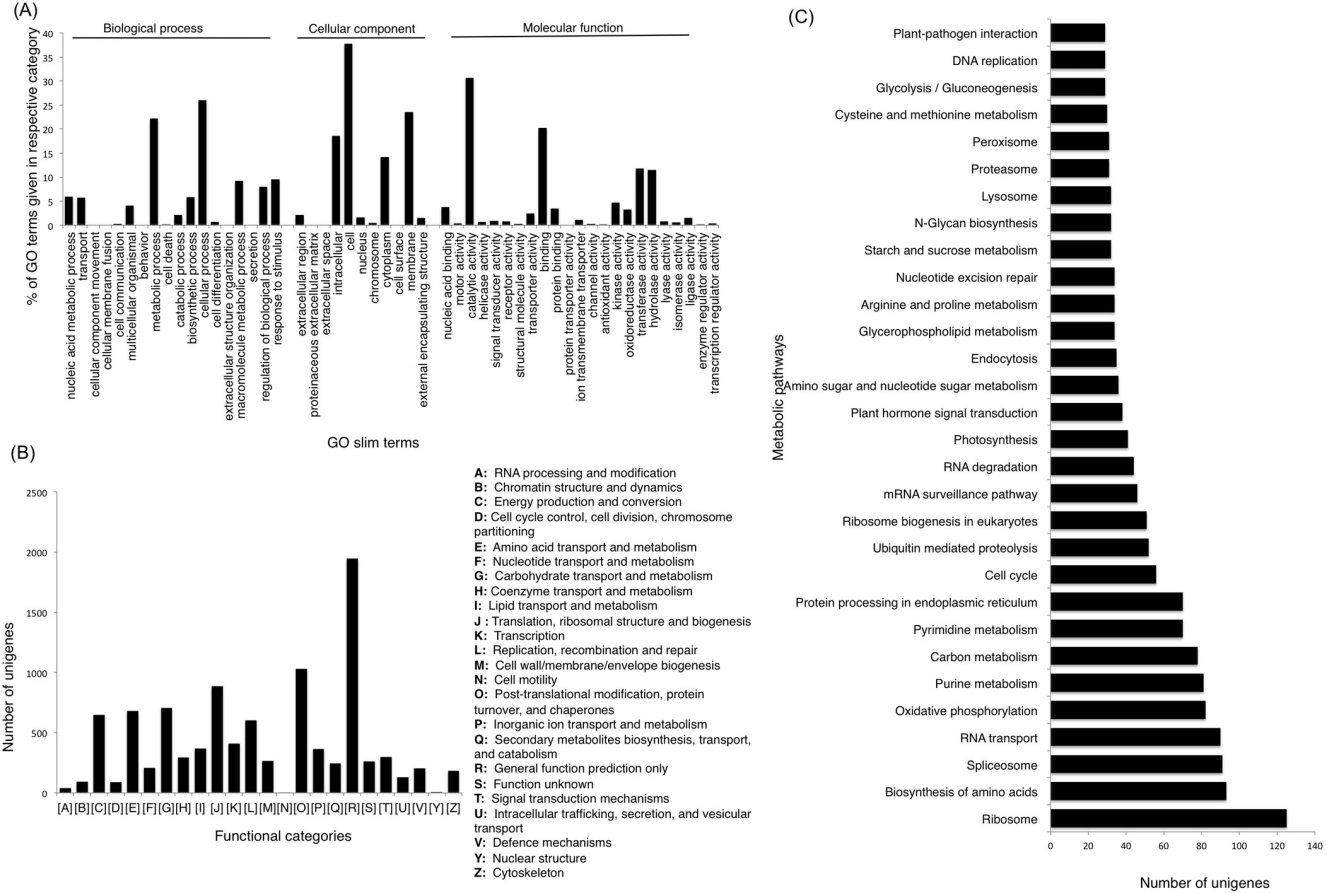
(A) GO annotations of chickpea root and nodule unigenes (B) Distribution of unigenes in different orthologous groups according to COG database (C) Distribution of unigenes into biological pathways using KEGG.

To assign putative function to chickpea transcripts, they were annotated using different strategies i.e. Gene Ontology (GO) based, Cluster of Orthologous Groups (COG) based and Kyoto Encyclopedia of Genes and Genomes (KEGG) pathway assignment (S2 Table). Transcripts were first subjected to BLASTX search against the non-redundant and annotated protein sequences present in UniProtKB/SwisProt database. Of the 83,405 assembled unigenes, 32,429 showed a significant match with the UniProtKB/SwisProt database. Gene Ontology terms were assigned to the transcripts (Fig 1A), which resulted into assignment of 5,299 GO terms which were further classified into three principle categories i.e. Biological process, Molecular function and Cellular component. As one GO term can be assigned to multiple transcripts and single transcript can have multiple GO terms, 25,877 sequences were assigned 2,897 GO terms under biological process category, 26,634 sequences were assigned 1,818 GO terms under molecular function category and 15,410 sequences were assigned 584 GO terms under cellular process category. In the biological process category most of the transcripts were associated with ‘cellular process’ followed by ‘metabolic processes’ and ‘response to stimulus’. Similarly in the cellular component category highest number of transcripts were associated with ‘cell’ followed by ‘membrane’ and ‘intracellular’ categories and in the molecular function category the largest number of transcripts were grouped in ‘catalytic activity’ followed by ‘binding’ and ‘transferase activity’ (Fig 1A).

The transcripts were also classified into protein families based on COG analysis which assigned 9,982 putative proteins to 99 orthologous groups which were further classified into 24 families of orthologous groups (Fig 1B). Out of the 24 functional categories, maximum number of putative proteins could be classified under categories 'General function prediction only' (1947) followed by ‘Post-translational modification protein turnover and chaperones’ (1030), ‘Translation, ribosomal structure and biogenesis’ (886) 'carbohydrate transport and metabolism' (707) and ‘Amino acid transport and metabolism’ (682). A number of transcripts were also classified in categories like 'cell cycle control, cell division and chromosome partitioning', 'cell wall and membrane biogenesis’, 'nucleotide transport and metabolism' and 'signal transduction mechanisms'.

To further understand the biological role of these transcripts, and to know the metabolic pathways they work in, transcripts were mapped online by KEGG Automatic Annotation Server (KAAS) (http://www.genome.jp/tools/kaas/) database. It revealed 24,489 unigenes to be involved in 323 predicted KEGG metabolic pathways. Highest numbers of unigenes were assigned to pathways related to ribosome (125), biosynthesis of amino acids (93), spliceosome (91), RNA transport (90), and oxidative phosphorylation (82). A number of unigenes were also assigned to pathways related to protein processing, carbon metabolism, cell cycle control and plant hormone signal transduction. Genes were also represented in NOD- like receptor signaling (4), calcium signaling (7) and nitrogen metabolism (11) pathways (Fig 1C).

### Differential gene expression analysis of genes during root nodulation

In order to determine the levels of expression of the unigenes involved in root nodulation in *C*. *arietinum*, the filtered reads from the root nodule transcriptome were mapped onto the assembled transcriptome of 83,405 unigenes. We found 64.3% unigenes to be expressed in uninfected root, 50.7% at early stage, 66.5% at middle stage and 76.3% at late stage of root nodulation. Differential gene expression analysis, using DEGseq package [39] in R showed a total of 6,186 unigenes to be differentially expressed. Out of these, 3,760 unigenes were found to be differentially expressed in nodule tissue with respect to control root tissue. A total of 935, 117 and 2707 unigenes were found to be differentially expressed in the case of early, middle and late stages respectively, when compared to uninfected root. Further 928, 3787 and 2818 unigenes were found differentially expressed between early and middle, early and late and middle and late stages respectively.

**Fig 2:**
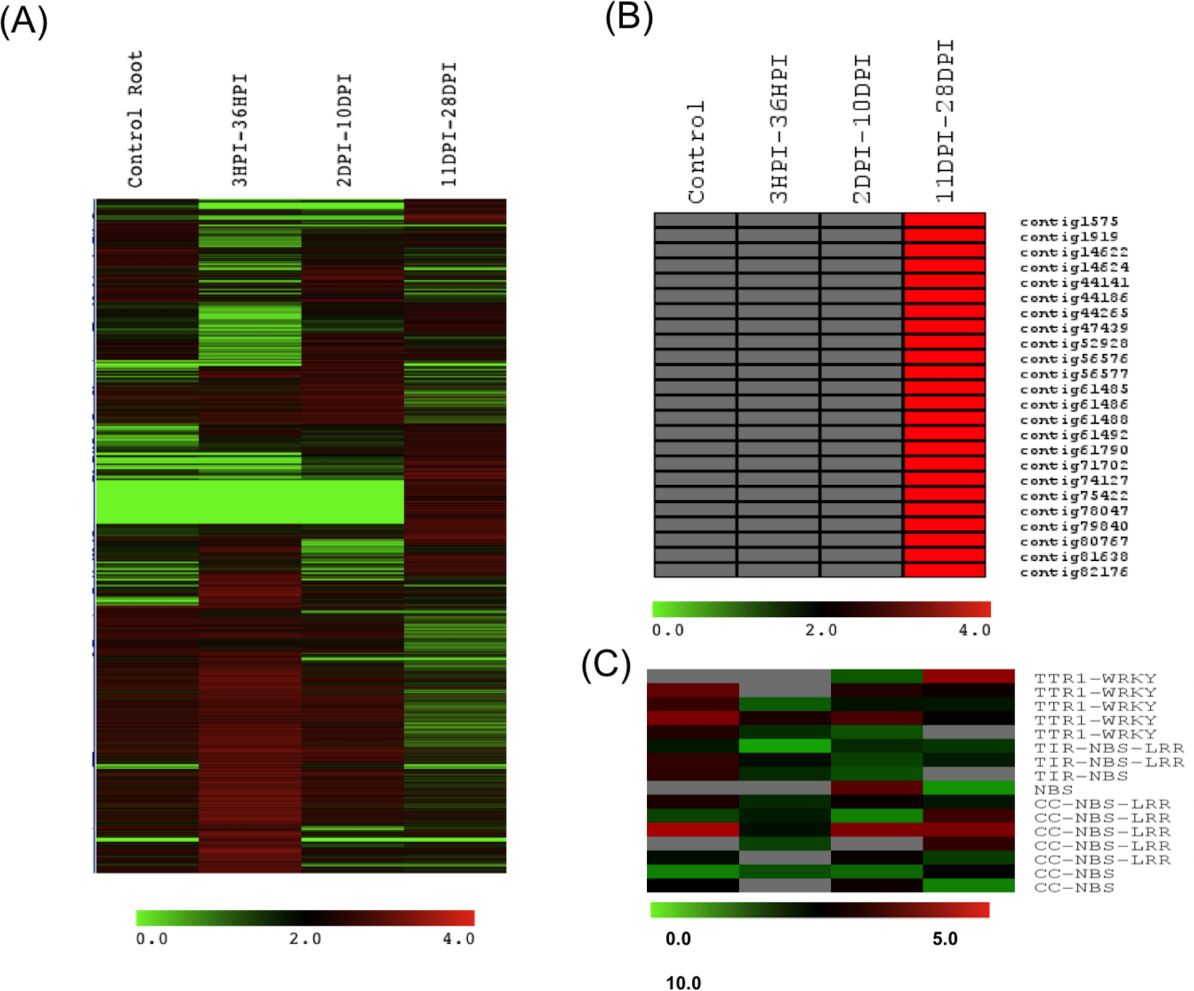
(A) Heatmap representation of differentially expressed unigenes in root and three stages of root nodule development. (B) Heatmap of nodule-specific cysteine-rich (NCR) peptides showing their expression during late stage of root nodulation. (C) Heat map of R-genes showing their expression pattern during root nodulation.

A heat map of differentially expressed transcripts was plotted using log_2_ transformed RPKM values (Fig 2A). It was observed that most of the differentially expressed unigenes in the early stages encoded peroxidases, GDSLesterase/lipase, defensin like protein, glutathione S-transferase, LRR receptor-like serine threonine-protein kinase, rch1-like, cell differentiation protein and some transporters including aquaporin and cyclin dependent kinase. Moreover, genes for proteins like Cytochrome p450, disease resistance protein rga4, β-amyrin, kinesin-like calmodulin-binding, ethylene responsive transcription factor, methyltransferase like protein and ABC transporters were seen to be preferentially expressed in the middle stage while genes for leghemoglobin, nodulins, nodule cysteine rich peptides, glutamate binding protein and some transporters i.e. bidirectional sugar transporter sweet10-like, peptide transporters, lipid transporters, ammonium transporter, nitrate transporters etc. had higher expression in the late stage (S3 Table).

The unigenes were further subjected to K-means clustering followed by figure of merit analysis (FOM) to classify differentially expressed genes in 20 clusters (S3 Fig). Cluster 3 showed 136 unigenes to be overall up regulated whereas cluster 6 showed 265 unigenes having a gradual increase in expression during nodule development. Cluster 9 showed 144 unigenes showing higher expression in early stage and cluster 1 represented 126 unigenes that were up regulated in early as well as in late stage of nodulation. Cluster 13 was represented by 271 unigenes which were up regulated in middle stage and cluster 5 showed 656 unigenes having higher expression in late stage (S4 Table). Each of the stages of nodule development was characterized by differential expression of genes belonging to various biological processes and molecular functions. Early stage showed highest number of unigenes involved in oxidation-reduction process followed by many important processes such as cellular biosynthesis, transport, cell cycle, cell differentiation and signal transduction. In middle stage maximum numbers of unigenes were categorized in organic substance metabolic process and primary metabolic process. Unigenes belonging to biosynthetic process, signal transduction, regulation of biological process, cellular component biogenesis and assembly and cell wall organization and assembly were also found differentially expressed. Later stage was represented by abundance of unigenes belonging to macromolecule metabolic process, organic substance biosynthesis process followed by transport, nitrogen compound metabolic process and carbohydrate metabolic processes. Other important categories were response to oxygen-containing compounds, symbiosis, encompassing mutualism through parasitism, response to oxygen levels, tetrapyrrole metabolic process and different transport processes such as carbohydrate transport, amide and amine transport, oxygen transport and hormone transport. Some unigenes belonging to telomere capping, glutamate biosynthetic process, protein folding, transcription regulation and anatomical structure development were found gradually increasing during nodule development.

“Nodule-specific cysteine-rich” (NCR) peptides are the recently discovered defensin like peptides that induce endoreduplication in rhizobia leading to differentiation of bacteria to functional bacteroids [43]. A homology-based search using *M*. *truncatula* NCR peptides was performed in order to identify their homologs in chickpea. This resulted in identification of 24 NCR peptides in root nodule tissue. Average length of NCRs were found to be 66 amino acids or 602 nucleotides and varied in size from 29–193 amino acids or 116–1659 nucleotides. An *in-silico* expression analysis of the NCR peptides from the chickpea nodule transcriptome showed that these genes had preferential expression in the later phase of nodule development (Fig 2B, S5 Table). Furthermore, 16 R genes were found differentially expressed during root nodulation and most of them belonged to CC-NBS-LRR family followed by TTR1-WRKY, TIR-NBS-LRR and CC-NBS. Of these, 11genes were found to be down regulated during root nodulation with the least expression being detected during early stage of nodule development (Fig 2C, S6 Table).

**Fig 3:**
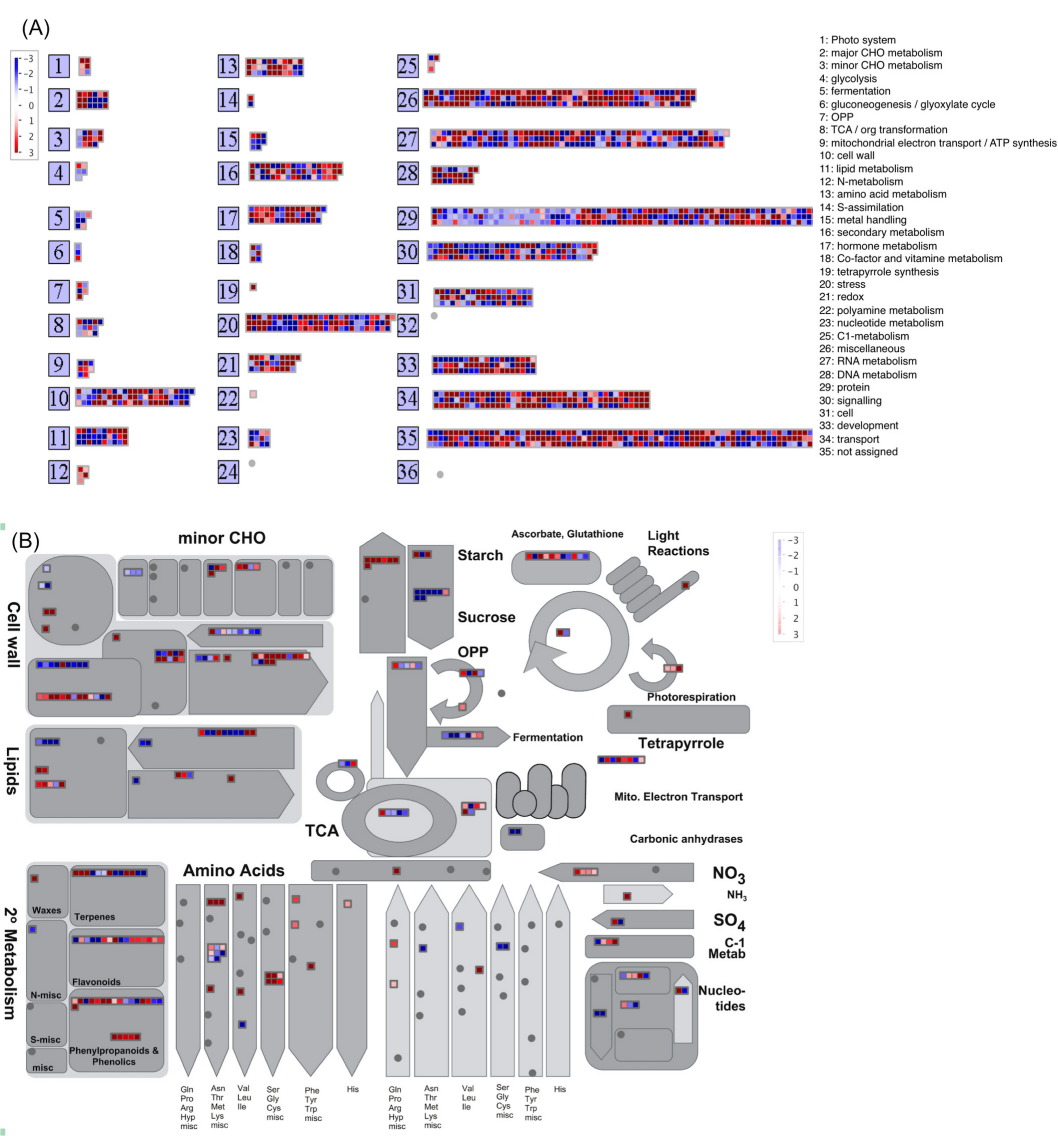
Metabolic pathways analysis of differentially expressed unigenes using MapMan(A) Bin wise distribution of unigenes (B) Distribution of unigenes in different metabolic pathways. Genes with upregulation in nodule tissue are shown in red and genes with reduced expression in nodule are blue.

In order to identify metabolic pathways preferentially expressed during root nodule development, differentially expressed chickpea transcripts were compared with *M*. *truncatula* protein sequences and searched against metabolic pathways profile present in MapMan [41]. Differentially expressed genes were seen to be distributed in many primary as well as secondary metabolic pathways (Fig 3). Primary metabolic pathways were majorly represented by cell wall biogenesis and degradation (BIN 10, 67 transcripts), plant hormone synthesis (BIN 17, 43 transcripts) tetrapyrrole synthesis (BIN 19, 1 transcript) nitrogen metabolism (BIN 12, 5 transcripts), amino acid metabolism (BIN 13, 32 transcripts), cell signaling (BIN 30, 97 transcripts), cell organization and cytoskeleton (BIN 31, 57 transcripts) and various transport systems (BIN 34, 125 transcripts) i.e. sugar transport, plant hormone transport, ion transport etc. Secondary metabolic pathways were mainly represented by flavonoids and phenols biosynthesis (BIN 16, 53 transcripts) (Fig 3A). In general, a number of transcripts were found to have preferential expression during various metabolic pathways such as cell wall biosynthesis, secondary metabolism, degradation of amino acids, starch biosynthesis, ascorbate and glutathione biosynthesis (Fig 3B).

### Mining of nodule specific transcripts

The control root (uninfected) and nodule were found to express 53,408 and 78,737 unigenes respectively with 48,740 unigenes in common. A comparison revealed 4,668 and 29,997 unigenes to be specifically expressed in uninfected root and nodule tissues respectively. In order to identify transcripts specifically involved in the process of nodulation, a comparison of the nodule transcriptome was done with the publicly available chickpea transcriptomes of tissues such as chickpea shoot, root, mature leaf, flower bud and young pod [36](http://www.nipgr.res.in/ctdb.html) and with the transcriptome of chickpea seed [25]. A TBLASTX search was done using chickpea nodule transcriptome as query. This alignment revealed 5,907 unigenes that did not find a match with any of the transcripts reported in the aforementioned databases. These unigenes were therefore considered to be putative nodule specific transcripts and have been referred to as such in subsequent analysis. Of these 5,907 nodule specific unigenes, only 649 could be assigned annotations (S7 Table) and the remaining 5,258 were predicted to be putatively novel nodule transcripts.

**Fig 4:**
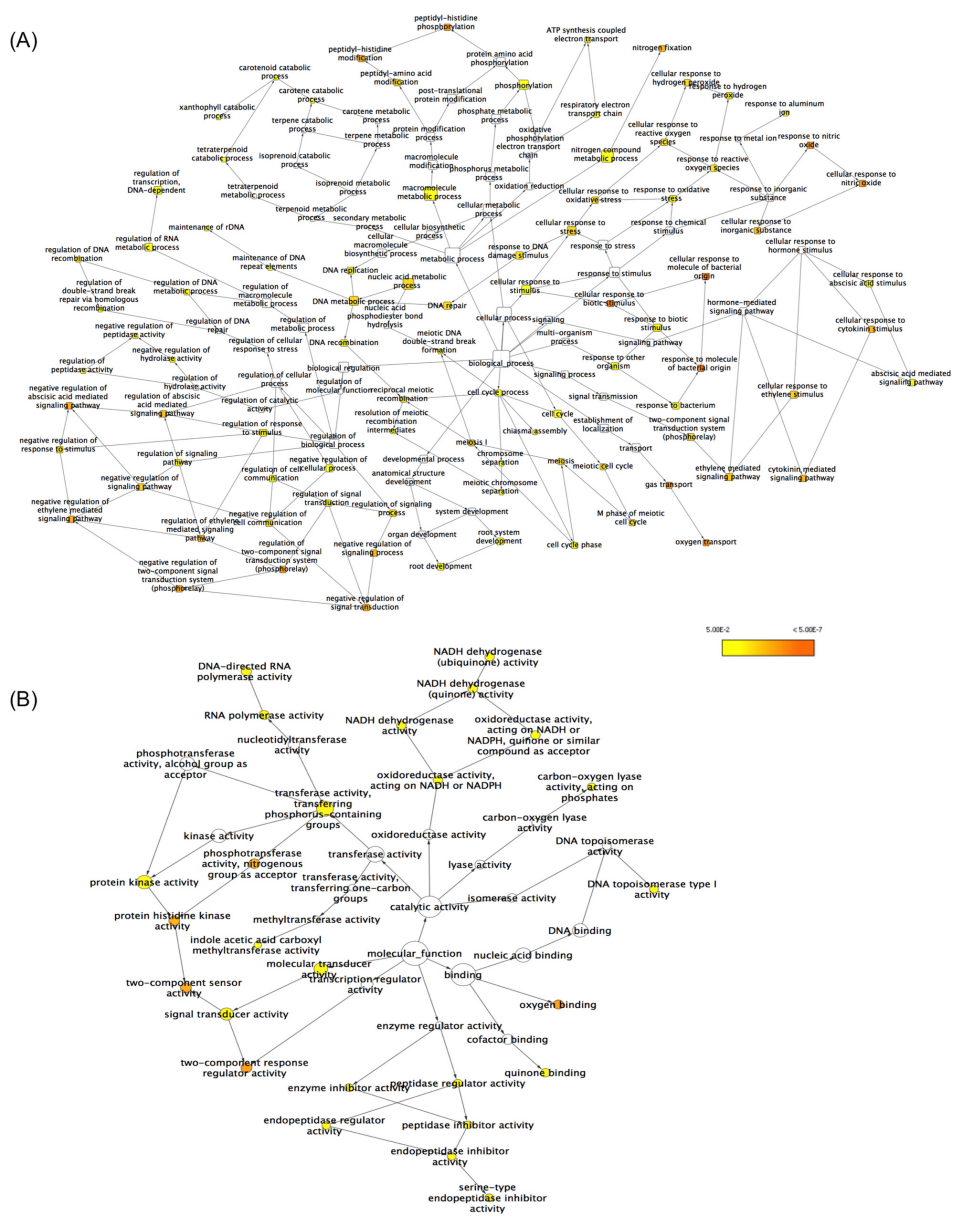
GO enrichment of putative nodule specific transcripts of chickpea using BiNGO 2.44 (A) Biological processes and (B) Molecular functions. Node size is proportional to the number of transcripts in each category and colors shaded according to the significance level; yellow depicting low significance levels and red depicting higher significance levels.

Further, to identify the major functional categories of the nodule specific transcripts GO enrichment analysis in BINGO 2.44 was performed (Fig 4, S8 Table). Transcripts involved in various pathways occurring during development of root nodule such as response to bacterial stimuli, signal transduction, ion transport, cell wall biogenesis, organ development were found to be enriched. Moreover, transcripts involved in root nodule functioning, including oxygen binding and transport, nitrogen fixation, and transport of amino acids were also found to be enriched. Under Biological processes, cellular response to molecule of bacterial origin, cellular response to biotic stimulus, oxygen transport, regulation of two-component signal transduction system (phosphorelay), cytokinin mediated signaling pathway and nitrogen fixation were among the most significantly enriched. GO categories related to nucleic acid metabolism and cell cycle control were also found to be enriched. Under Molecular functions, GO terms associated with oxygen binding, two-component response regulator activity, RNA directed DNA polymerase, endopeptidase inhibitor activity, NADH dehydrogenase activity were most significantly enriched in addition to categories like RNA polymerase, DNA binding and transcription regulation activity.

Transporters play a very important role in development and functioning of the root nodule by mediating influx and efflux of different ions and metabolites between the two counterparts of symbiosis. Hence, a search of the putative nodule specific transcripts revealed the presence of 17 transporters. Important transporters specifically present in nodules were amino acid transporters, C4-dicarboxylate transporters, polyol transporters, ammonium ion transporters, ABC transporters, potassium ion transporters, calcium transporters etc.

### Transcription factors in chickpea root nodule development

**Fig 5:**
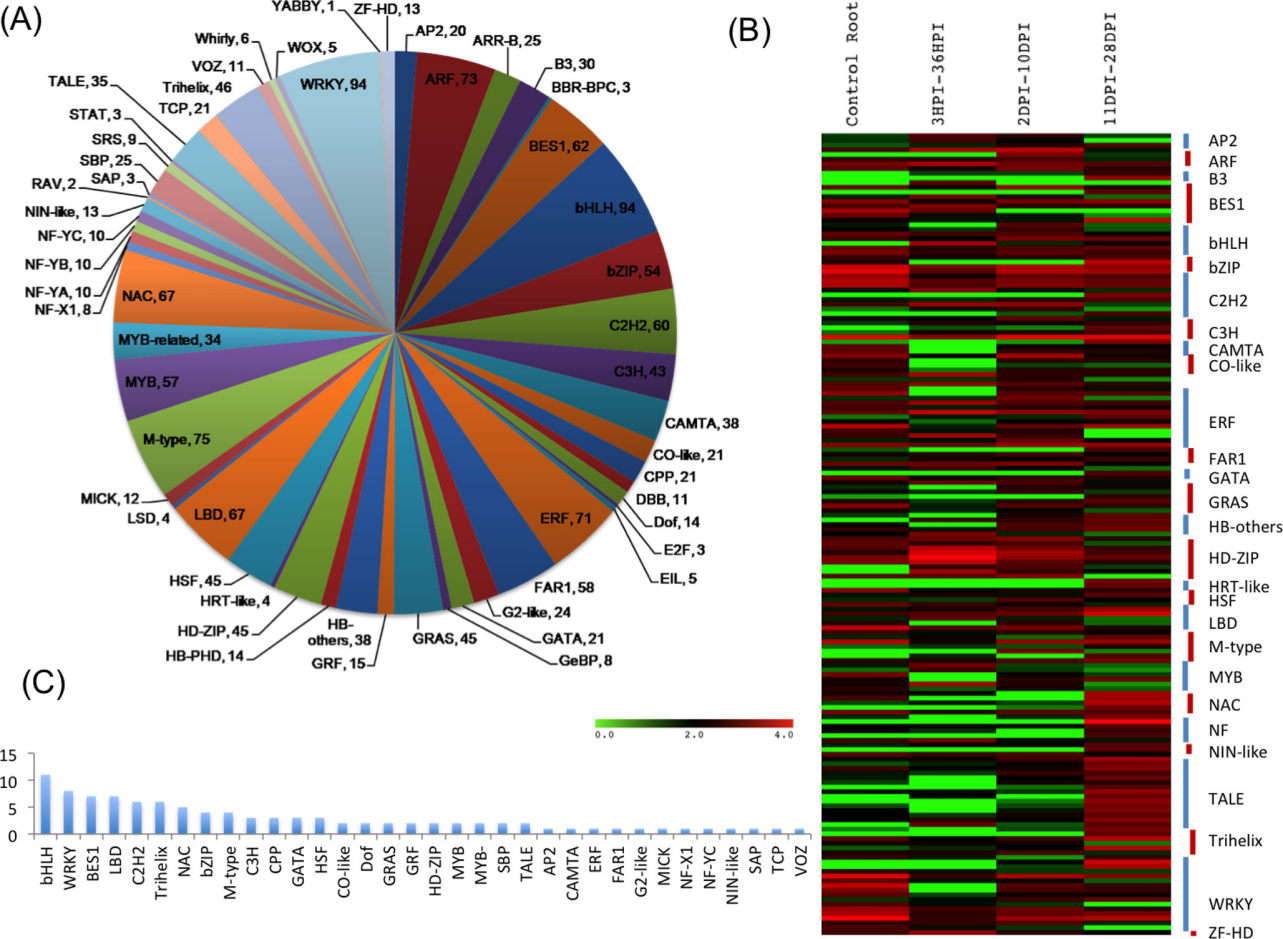
(A) Distribution of unigenes in TF families (B) Differential expression patterns of TFs in various stages of nodule development (C) Distribution of putative nodule specific TFs in different families.

Eukaryotes have developed a complex way of regulating expression of their existing genes through a group of proteins known as transcription factors (TFs) that directly affect their growth and development in a variety of ways. Peptide sequences of different TFs from five legumes (*Cajanus cajan*, *C*. *arietinum*, *G*. *max*, *L*. *japonicus* and *M*. *truncatula*) were downloaded from the Plant Transcription Factor Database (http://planttfdb.cbi.pku.edu.cn) and their HMM profiles were built using hmmbuild (HMMER 3.0, ftp://selab.janelia.org/pub/software/hmmer) and used to search the chickpea nodule transcriptome. Out of 83,405 unigenes, 1606 unigenes were identified to be TFs belonging to 55 known TF families (Fig 5A). The most abundant families were bHLH, WRKY, M-type, ARF and ERF families followed by LBD, NAC, BES1, C2H2 and FAR1. Moreover MYB, bZIP, GRAS, HD-ZIP and NIN-like families were also found in the dataset. The *in-silico* differential expression analysis of the 1606 TFs revealed that 171 TFs belonging to 40 families were differentially expressed during root nodule development (Fig 5B). Highest number of differentially expressed TFs belonged to WRKY family followed by TALE, ERF, C2H2 and HD-ZIP. Many TF families well known to be involved in root nodulation process such as ERF, GRAS and NIN-like were also found to be differentially expressed. Moreover, 100 unigenes belonging to 35 families of plant TFs were found specifically expressing in nodule tissues (Fig 5C), of which TFs belonging to bHLH family were found to be highest in number followed by the WRKY family.

### Validation of digital expression data

**Fig 6:**
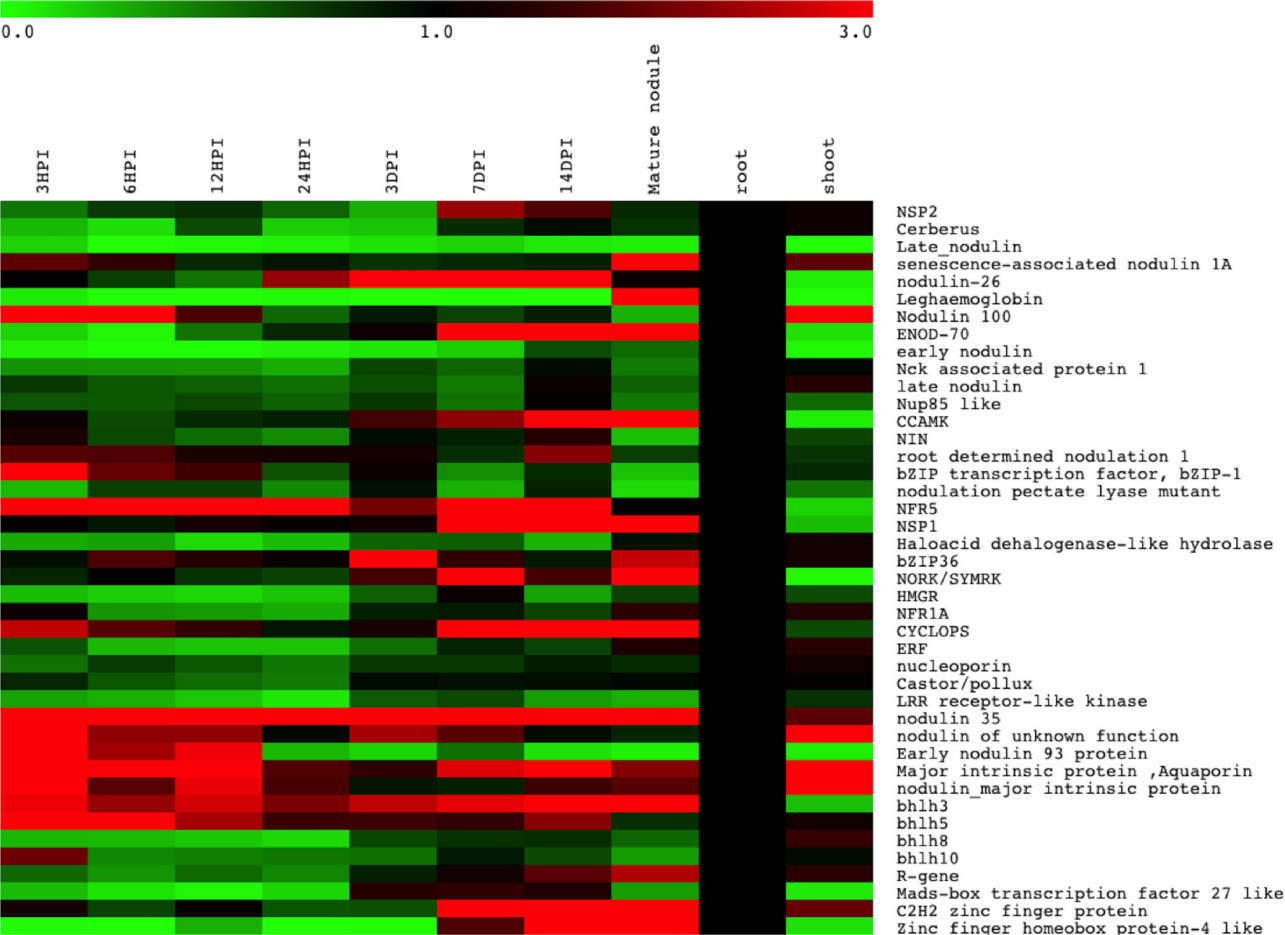
Differential expression pattern of unigenes by qRT PCR analysis.

To validate the RPKM based digital expression data, quantitative RT-PCR analysis was performed using 8 different developing stages of nodules, root and shoot tissues of chickpea. Most of the genes chosen for this analysis included those predicted earlier to be involved in root nodulation process in chickpea and hence, may serve to further validate our analysis. The list of primers used in qRT-PCR analysis is given in S9 Table. Real time analysis revealed that most of the genes, which were earlier predicted to be involved in nodule functioning [19], showed preferential expression in different stages of nodule development (Fig 6). These included the genes for NOD factor receptors, bZIP transcription factor, early nodulin 93 (*ENOD-93*), *NSP1*, *NSP2*, *CCaMK*, *LjCYCLOPS* homologue, *aquaporin*, a nodulin of unknown function and some other transcription factors which were found to be upregulated in early to middle stages of nodule development. Later stage of root nodule showed up regulation of *leghemoglobin*, *senescence associated nodulin*, *aquaporin* and a R-gene. This analysis confirmed that the pattern of gene expression in *in-silico* as well as that revealed by qRT PCR analysis were similar thereby implying that RPKM based digital expression analysis was a reliable method for determining differential gene expression.

### Identification of SSRs in chickpea root nodule transcriptome

Simple Sequence Repeats (SSRs) are nucleotide repeats present in DNA sequences and are known to serve as excellent molecular markers for genotyping purposes. Therefore, the transcripts were searched for the presence of microsatellite motifs using MISA (MIcroSAtellite) tool (http://pgrc.ipk-gatersleben.de/misa). A total of 11,318 SSR motifs were identified in 8,724 transcripts of *C*. *arietinum* with the frequency of one SSR per 2.86kb. Tri-nucleotide repeats were found to be most abundant (44.71%), as expected in genic SSRs, followed by tetra-nucleotide repeats (25.64%) and di-nucleotide repeats (13.27%). In the case of tri-nucleotide SSRs the most abundant class was the GAA/TTC followed by AAT/ATT and CAA/TTG (Table 3). The flanking sequences of the SSRs were used to design 6,950 pairs of primers (S10 Table) representing 5,398 transcripts for use as molecular markers.

**Table 3:** Numbers and distribution of SSRs in chickpea root and nodule tissue.

Total number of sequences examined	83,405
Total size of examined sequences (bp)	32,393,843
Total number of identified SSRs	11,318
Number of SSR containing sequences	8,724
Number of sequences containing more than 1 SSR	1,775
Number of SSRs present in compound formation	721
Distribution of SRRs across different repeat type classes	
2 (dinucleotides)	1,503
3 (trinucleotides)	5,061
4 (tertanucletides)	2,903
5 (pentanucleotides)	934
6 (hexanucleotides)	917

## Discussion

Symbiotic association between chickpea and *M*. *ciceri* is an economically important phenomenon leading to fixation of large quantities of atmospheric nitrogen, which occurs in specialized organs called ‘nodules’. The advent of NGS technologies has facilitated the deep transcriptome analyses of cells, tissues and organs. Transcriptome has been utilized to analyze the symbiosis in nodules of *L*. *japonicus* [44], *M*. *truncatula* [28] and *G*. *max* [31]. However the transcriptomic analysis of developing nodules in chickpea has not been carried out even though the transcriptomes of all other tissues have been reported [25, 36]. Hence this study was designed to gain an insight into the process of nodulation in chickpea by using the NGS technology to sequence, assemble and analyze the chickpea root nodule transcriptome at various stages of development. Reference based assembly was carried out as the chickpea genome was available [19]. In a reference-based assembly approach, artifacts can be eliminated as any sequenced read is assembled only if it aligns somewhere in genome. In addition, rare or low abundance transcripts can be assembled and new transcripts can also be searched. Additionally, reference based assembly also ensured removal of reads arising from *M*. *ciceri* bacterial RNA contamination. The assembly was validated by aligning it to chickpea genome sequence and comparison with other known protein databases. Approximately 72% of assembled transcripts got a match when compared to gene models predicted in chickpea genome sequence, which represent 63.49% of predicted gene models [19]. On the other hand 73.64% transcripts found a match in at least one of the five proteome databases. These results validated the quality of the assembly and suggested wide coverage of gene models in the transcriptome. Only 38.88% of chickpea transcripts found a match in UniProtKB/Swiss-Prot database. This could be attributed to the low representation of functionally annotated genes in UniProtKB/Swiss-Prot database. GO annotation of transcriptome revealed that the assembled transcripts had diverse functions. Moreover, COG and KEGG annotation also suggested that they were involved in many metabolic pathways. Overall, the annotation implied that the transcriptome had extensive coverage, encompassing most of the genes involved in chickpea root nodulation.

Analysis of the differentially expressed genes across the early, middle and late stages with respect to control (uninfected) root helped in identifying genes of nodule specific metabolic pathways which were predominantly stage specific. Early and middle stages of nodule development demonstrated up-regulation of genes associated with cell signaling, transcriptional control, cell differentiation and some antioxidant activity whereas the later stage was characterized by the presence of many structural and functional proteins, some of which have been functionally characterized for establishing their role in nodule development. It has been seen that early response to infection is similar for pathogenic and symbiotic bacteria such as production of reactive oxygen species (ROS) [45]. ROS act as negative regulators of nodulation [46] by restricting the entry of rhizobia into the plant. Genes such as Glutathione-S-transferase (gst) and peroxidases are involved in the metabolism of ROS and up-regulation of these ROS metabolizing genes was observed during early stage of rhizobium infection in chickpea, which clearly indicated their involvement in establishing successful infection [47]. Moreover, down regulation of ‘Resistance genes’ during root nodulation was also observed which supported the notion that defense response is inhibited during root nodulation for successful establishment of symbiosis [48]. Cell wall dynamics plays an important role in infection and development of nodules. Hence, transcripts related to cell wall biogenesis (cellulose synthesis and hemi-cellulose synthesis) and cell wall degradation (pectate lyase) were also found to be up regulated. Pectate lyase which is induced in plant cells in response to NOD factor, degrades the cell wall locally to form the infection thread and is a prerequisite for bacterial infection and formation of functional nodule [49].

Upregulation of many p450 family genes has been demonstrated in response to nodulation [50] and similar results were also obtained in this study. β-amyrin synthase was found to be upregulated, which has been reported to play an important role in root epidermal patterning [51] and its over-expression has been shown to enhance root nodulation in *M*. *truncatula* [52].

Analysis of transcriptome from the later stage of nodule development revealed up regulation of different transcripts, suggesting their involvement in the biological functioning of the mature nodule such as nitrogen fixation and assimilation. Genes such as glutamine synthetase (GS) and glutamate dehydrogenase (GDH) are known to be involved in nitrogen metabolism and responsible for the assimilation of fixed nitrogen by the plant [53] and were found to be up regulated in mature root nodule. Moreover, role of leghemoglobin in nodule functioning [54] is well established as it is involved in maintaining oxygen homeostasis and hence its upregulation in late stage in our data validated our study. NCR peptides are important for bacteroid differentiation and formation of functional root nodules and are specific to the legumes of the Inverted Repeat-Lacking Clade (IRLC) in the *Leguminosae* family, which includes chickpea [55]. It has been established that the bacteroid differentiation stops completely after blocking the delivery of symbiotic peptides to the bacteroids [56]. Significant expression of the NCR peptides in the mature chickpea nodules suggested their role in bacteroid development, which needs to be further investigated.

An important objective of this study was identification of genes specifically involved in root nodulation events in chickpea. A set of 5907 nodule specific unigenes, which were present only in root nodule tissue, when compared to other chickpea tissues, were identified, of which 649 could be annotated. This low frequency of annotation was probably due to the presence of putative novel transcripts that could not find any match in the existing Non Redundant database of NCBI. GO enrichment of putative nodule specific transcripts revealed their involvement in cytokinin mediated signaling, two component signal transduction, oxygen binding and transport, cell cycle control and transcriptional regulation etc., which are implicated in the crucial process of nodule development. Moreover, exclusive presence of transcripts involved in nitrogen fixation, cell communication and cell differentiation in the chickpea root nodule clearly suggest the biological significance of these processes in the event of nodulation.

Another class of putative nodule specific genes, which attracted our interest, was the transporters. The importance of transporters cannot be denied, as they are the proteins that work as a bridge between the plant and the rhizobia, making symbiosis a feasible phenomenon. Primary exchange between roots and rhizobia involve exchange of sugar (reduced carbon) for reduced nitrogen (mainly in the form of NH4^+^) [53]. As C4-dicarboxylates are the principal source of reduced carbon for the bacteriods in root nodule, hence C4-dicarboxylate transporters are extensively involved in feeding bacteriods. Our results revealed the presence of 4 nodule specific malate transporters in chickpea, which may be putatively involved in supplying carbon source to bacteriods [57]. Another significant observation was the presence of specific ammonium ion transporters in chickpea. Earlier studies of soybean ammonium transporter have shown them to be involved in transfer of fixed nitrogen from bacteroid to host [58]. Presence of ATP Binding Cassette (ABC) type transporters was also justified since they are known to be involved in secretion of flavonoids [59] and hence play a key role in helping rhizobia to recognize its host plant and initiate infection. Moreover, presence of the calcium ion transporter that plays a role in NOD factor signaling was also significant and it would be interesting to explore the roles of some of the putative nodule specific transporters that were identified in the chickpea nodule transcriptome.

Transcription Factors (TFs) play an important role in orchestration of the metabolic pathways by regulating gene expression of related encoding enzymes. Annotation of a large number of transcripts as transcription factors in our data indicated a high degree of transcriptional regulation during nodule development. Occurrence of transcripts belonging to the RWP-RK (NIN-like), bHLH, NAC, WRKY GRAS and MYB transcription factor families in our transcriptome was significant, as some members of these families have an established role in root nodulation. The Nodulation Inception Protein (NIN) belonging to RWP-RK family of transcription factors is known to play a key role in nodule initiation [60]. Earlier studies have shown that NSP1 of GRAS family is essential for all NOD factor induced changes in gene expression [10], whereas NSP2 also plays a significant role in NOD signaling [11]. A transcription factor of the bHLH family (MtbHLH1) has been known to play a very critical role in vascular patterning and facilitating nodule to plant metabolic exchanges in *M*. *truncatula* [61]. A global analysis of transcriptional reprogramming during *M*. *truncatula* root nodule development has shown up-regulation of many transcription factors [28], which was very similar to the results obtained in our study.

In conclusion, this study was undertaken with the objective of in-depth analysis of the root nodulation event in chickpea. The assembly and annotation of the chickpea root nodule transcriptome indicated a good coverage of the genes reported in chickpea genome [19]. A number of genes that have been reported to play important regulatory roles in root nodulation were also found to be significantly expressed in the chickpea root nodule transcriptome, thereby corroborating the various analyses presented in this study. Digital expression analysis also showed that a number of TFs play an important role in the process of nodulation. In addition, this study highlighted the involvement of NCR peptides and transporters in the process of nodule formation and provides valuable foundation for further characterization of these genes to study their role in development of chickpea root nodules. Therefore, the high quality of dataset provided here will serve as a foundation for developing a functional genomics platform for investigating the chickpea-rhizobia symbiosis leading to improvement of crop chickpea.

## Supporting Information

S1 Data Assembly of root and nodule transcriptome. (ZIP)

S1 Fig Size distribution of assembled unigenes; X axis shows size interval and Y axis shows number of transcripts in a class. (JPG)

S2 Fig Validation of transcripts by amplification by PCR. (JPG)

S3 Fig k-means clustering of differentially expressed unigenes. (JPG)

S1 Table Comparison of chickpea transcriptome to proteomes of *A*. *thaliana*, *G*. *max*, *L*. *japonicus*, *M*. *truncatula* and *P*. *trichocarpa*. (XLS)

S2 Table GO, COG and KEGG annotation of chickpea root nodule unigenes. (XLSX)

S3 Table Annotation of significantly differentially expressed unigenes. (XLSX)

S4 Table k-means clustering of significantly differentially expressed genes. (XLSX)

S5 Table RPKM values and amino acid sequences of NCR peptides. (XLSX)

S6 Table Log2 normalized RPKM values and nucleotide sequences of differentially expressed R-genes. (XLSX)

S7 Table Annotation of putative nodule specific unigenes. (XLSX)

S8 Table GO enrichment of putative nodule specific unigenes. (XLSX)

S9 Table List of primers used in qRT PCR analysis. (XLSX)

S10 Table List of primers generated for SSR markers. (XLSX)
