# Supplementary figures and images for: Dissecting the Root Nodule Transcriptome of Chickpea (Cicer arietinum L.)
Source: PLoS One. 2016 Jun 27;11(6):e0157908. doi: 10.1371/journal.pone.0157908 (PMC4922567; doi:10.1371/journal.pone.0157908)

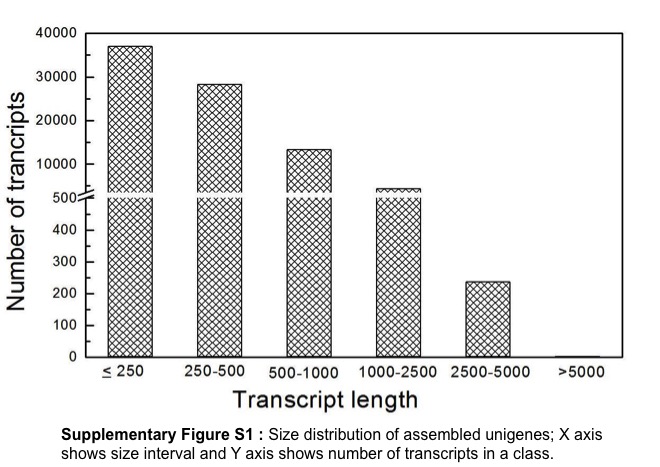

Supplement: S1 Fig — (JPG) [file pone.0157908.s002.jpg]

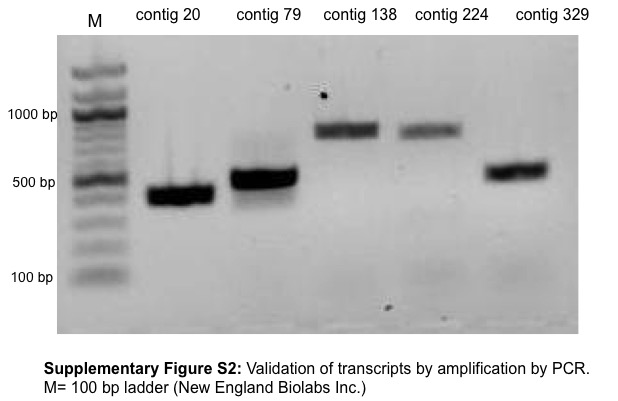

Supplement: S2 Fig — (JPG) [file pone.0157908.s003.jpg]

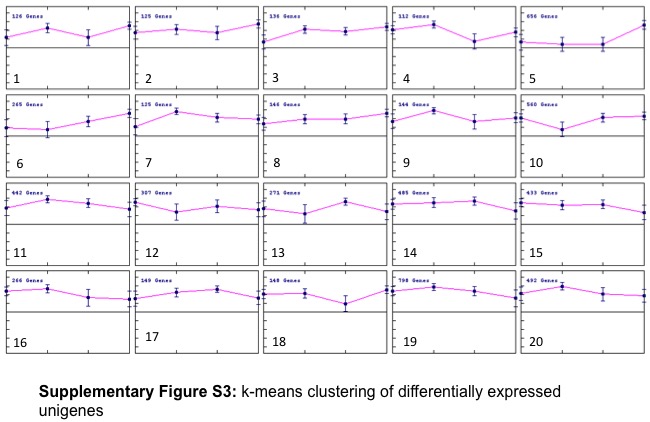

Supplement: S3 Fig — (JPG) [file pone.0157908.s004.jpg]
